# Factors associated with total cholesterol and blood glucose levels among Afghan people aged 18–69 years old: Evidence from a national survey

**DOI:** 10.1371/journal.pgph.0004079

**Published:** 2025-03-26

**Authors:** Giti Azim, Hosna Hamidi, Mohammad Shafi Azim, Bahara Rasoly, Mohammad Hasher Azim, Sultan Ahmad Halimi, Mohamed Mostafa Tahoun, Jamshed Tanoli

**Affiliations:** 1 World Health Organization Country Office, Kabul, Afghanistan; 2 Mental Health Hospital, Kabul, Afghanistan; 3 UNICEF, Kabul, Afghanistan; 4 Geomatika University, Kuala Lumpur, Malaysia; 5 Kabul University of Medical Sciences, Kabul, Afghanistan; 6 High Institute of Public Health, Alexandria University, Alexandria, Egypt; Universiti Malaya, MALAYSIA

## Abstract

The objective of this study was to determine the associated factors of total cholesterol (TC) and blood glucose (BG) levels in people aged 18-69 years old in Afghanistan. This was an analytical cross-sectional study using data from the National Survey of Non-Communicable Disease STEPs 2018 (NCD STEPS) in Afghanistan. The total sample size in the original study was 3,972 and a multi-stage cluster sampling method was used. Total cholesterol and blood glucose were the outcome variables for this study; simple and multiple linear regression was performed to find the associated factors for the outcome variables using a designed-based modeling incorporating sampling techniques and weights. The result of univariate linear regression analysis indicates that age, marital status, hypertension, and BMI are positively associated with TC and BG levels while education, salt intake, and any type of physical activity are negatively associated with TC and BG levels (p-values<0.05). Each year of age increases TC by 0.42 mg/dl and BG by 0.48 mg/dl; ever-married individuals have higher TC (21.8 mg/dl) and BG (8.8 mg/dl) levels; hypertension increases TC by 16.8 mg/dl and BG by 14.5 mg/dl; and higher BMI is associated with increased TC (1.3 mg/dl) and BG (0.9 mg/dl). Moreover, multivariate analysis using multiple linear regression indicates the same result; however, the results of marital status and gender are not significant with BG level and results of education levels, salt intake and any type of physical activity are not significant with TC levels. The finding of this study shows that total cholesterol and blood glucose increase in people of older age categories, married people, people with hypertension, overweight, and obesity; while decreases in people with higher education categories, people who always take salt, and people who do physical exercise.

## Introduction

The blood lipids are lipids or fats in the blood which can be free or bound to other molecules. Blood lipids include four categories of lipoproteins; 1) Total Cholesterol (TC); 2) High-density Lipoprotein (HDL); 3) Low-density Lipoprotein (LDL); and 4) Triglycerides (TG). Total Cholesterol includes both Low-density Lipoprotein (LDL) and High-density Lipoprotein (HDL) cholesterol. Through lipid test from serum, the amount/level of blood cholesterol and blood sugar are measured in milligrams per deciliter (mm/dl) or millimoles per liter (mmol/L); however, within 12 hours before the test, the person should not eat or drink anything other than water [1].

Blood glucose is the main sugar in the body and the source of energy for body activities [2]. Higher fasting blood glucose (hyperglycemia) in two separate tests indicates that a person has Diabetes while low blood sugar will indicate hypoglycemia which will cause dizziness, sweating, palpitation, and blurred vision [3]. Through diabetes test from serum, the amount/level of blood sugar is measured in milligrams per deciliter (mm/dl) or millimoles per liter (mmol/L); however, within 8 hours before the test, the person should not eat or drink anything other than water [3].

The normal level of total cholesterol differs in adults and children; for adults, total cholesterol levels less than 200 mg/dl are considered normal; 200 – 239 mg/dl is considered borderline while TC level at or higher than 240 mg/dl is considered high [4]. As for fasting blood glucose, the normal range is 99 mg/dl or below, 100 – 125 mg/dl indicates a person has prediabetes, and BG levels 126 mg/dl or higher indicate having diabetes millitus [5].

The worldwide age-standardized crude estimate mean level of total cholesterol and fasting plasma glucose were 4.5 mmol/l (174.0 mg/dl) estimated in 2018 [6] and 5.5 mmol/l (99.1 mg/dl) estimated in 2008 [7]; also, the NCD STEPs survey which was also conducted in 2018, estimated the mean level of total cholesterol and blood glucose to be 148.1 mg/dl and 87.8 mg/dl respectively [8].

A cohort study on serum cholesterol, blood pressure, and mortality implications among 361,662 men which was conducted in 1986, found that coronary heart disease (CHD) mortality increased in higher than 20^th^ percentile of serum cholesterol (>181 mm/dl) [9]. High levels of blood cholesterol are a risk factor for cardiovascular diseases; also ischemic heart disease and non-fatal stroke are among its primary outcomes [10]. In another study among patients with systemic lupus erythematosus (SLE), total cholesterol was among the risk factors for renal deterioration [11].

As studies suggest, high total cholesterol is a major risk factor for cardiovascular diseases. The prevalence of raised total cholesterol (defined as ≥5 mmol) was nearly 40% at the global level in 2008; while it increased noticeably based on the income level of the countries. In low-income countries, around a quarter of adults had raised total cholesterol, in lower-middle-income countries this rose to around a third of the population for both sexes. In high-income countries, over 50% of adults had raised total cholesterol; more than double the level in the low-income countries [12]. Moreover, a cross-sectional survey in 35 low and middle-income countries found that the prevalence of high total cholesterol (≥240 mg/dl) was 7.1%. High total cholesterol was among the top 10 major contributors for global DALYs among three risks in 2015 [13] and the global age-standardized mean total cholesterol was 178mg/dl [14].

High levels of blood glucose will cause diabetes and as per NCD survey, the prevalence of diabetes mellitus (≥126 mg/dl) was 9.2% in 2018. Based on the International Diabetes Federation, the global prevalence of diabetes was estimated to be 9.3% (463 million people) in 2019 while this estimate is expected to increase to 10.2% (578 million) by 2030 and 10.9% (700 million) by 2045. The prevalence is estimated higher in high-income countries (10.4%) than in low-income countries (4.0) [15]. However, based on the global burden of disease data from 1990 to 2019, the largest increase in Age Standardized Death Rate (ASDR) and age-standardized DALYs rate attributed to diabetes type 2 was reported in lower and middle-income countries (1.3% and 1.6% respectively) [16].

While there are lots of longitudinal studies on factors associated with total cholesterol and factors associated with blood glucose in other countries; no such studies have been conducted in Afghanistan, and since conducting longitudinal studies is difficult at this time frame considering the need for resources and current circumstance in the country; hence this study is aimed to determine the associated factors of total cholesterol and blood glucose using the evidence from the national NCD STEPs survey.

Recognizing the paramount importance of early detection of diseases and risk factors, this study offers significant contributions by identifying critical factors associated with total cholesterol and blood glucose levels among the Afghan population. By leveraging data from a national survey, this research underscores the necessity of targeted interventions and preventive measures to mitigate the risks of cardiovascular diseases and diabetes.

## Methodology

### Study design

The current study is an analytical retrospective cross-sectional study design using data from the National Survey of Non-Communicable Disease STEPs (NCD STEPS) in Afghanistan.

### Summary of the National NCD STEPs 2018

The STEPs 2018 survey, conducted from January 2018 to December 2019, aimed to assess non-communicable disease risk factors among Afghan adults aged 18-69 years. The survey used a multi-stage cluster sampling method to select a representative sample of 3,972 participants from 55 districts across Afghanistan. STEPS is the WHO’s recommended tool for surveillance of NCDs and their risk factors; the STEPS instrument (questionnaire) covers three different levels, or ‘steps’ of risk factor assessment: Step 1 (demographic and behavioral information); Step 2 (Physical Measurement – blood pressure, height, weight, and waist); and Step 3 (Receiving and testing participants’ urine and blood samples). Data was collected using tablets and uploaded to the eSTEPS platform.

### Inclusion and exclusion criteria

**Inclusion criteria:** Household permanent residents aged 18-69 who showed willingness to participate in the study.

**Exclusion criteria:** Temporary residents (residing for less than 12 weeks) of households aged 18-69 and those who refuse to participate in the study.

**Sample Size and Sampling:** The sample size in the original survey was calculated using the formula $n=Z^{2}\frac{P\left(1-P\right)}{e^{2}}$ to have reliable estimates for 6 strata that were 3 age groups (18-29, 30-44, and 45-69) in each of the sexes. Using a 95% confidence level, 5% margin of error, 0.5p, and 0.5q, the resultant sample size was 384. With a 1.5 design effect and 15% non-response rate the sample size was adjusted to 662 for each strata of the age-sex group. The adjusted sample size was multiplied by 6 age-sex groups (662*6) to get the final sample size of 3,972 household members.

In the original study multi-stage cluster sampling method was used; districts were used as primary sampling units (PSUs), villages were the secondary sampling units (SSUs), and households within districts as tertiary sampling units (TSUs). Then, within each household, data from a randomly selected eligible member was collected.

Based on the guidelines of the WHO, the total number of PSUs within a sampling frame should be greater than 100 among which 50-100 PSUs should be randomly selected. The total number of districts in 34 provinces of Afghanistan was 417. From 417 districts 55 districts were selected using the PPS method through the Stepwise-Approach XLs form. The total sample size was distributed proportionate to the size of the districts, then the sample size of the districts was divided by 15 (maximum number of the households to be interviewed within an Enumeration Area (EA)/village) and the number of villages/EAs within each district was calculated. Using the EPI sampling frame villages/EAs were selected within each district using PPS methodology. Within each village/EA the total number of the households were calculated, and it was divided by 15 to calculate the sampling interval. Using the sampling interval household within each village were selected through systematic random sampling methodology. Then, within each household all eligible members were listed, and one member was randomly selected from whom the data was collected.

In the original survey, data was collected using the STEPS Android App and uploaded automatically into the eSTEPS platform. Two visits were conducted for each individual for data collection; in the first visit demographic, behavioral, and physical measurement information was collected and in the early morning of the second visit blood (fingertip) and urine samples of the same respondent were collected in order to measure the fasting total cholesterol and fasting blood glucose levels. To generalize the study findings to the whole population, 2 different weightings were applied: weights for Steps 1 & 2 and weights for Step 3. Weights were calculated to adjust for the 3 aspects of the survey; 1) probability of selection (sample weight); 2) non-response (non-response weight); and 3) differences between the sample population and target population (population weight).

### Secondary analysis approach

Secondary analysis was chosen for this study due to several reasons. Firstly, it allows for the efficient use of existing data, which is particularly valuable given the resource constraints and current circumstances in Afghanistan. Secondly, secondary analysis enables the exploration of new research questions and hypotheses that were not the primary focus of the original survey. This approach is well-suited to address the research question of identifying factors associated with total cholesterol and blood glucose levels, leveraging the comprehensive dataset from the National Survey of Non-Communicable Disease STEPs 2018.

### Patient and public involvement statement

This study was a secondary analysis; thus, the patients and the public were not directly involved in study design or data collection. However, the findings of this study were presented in the FMIC Annual International Scientific Conference, allowing for dissemination to a broader audience, including healthcare professionals, researchers, and potentially interested members of the public. The presentation provided an opportunity for feedback and discussion, contributing to the relevance and impact of the research.

While direct involvement of patients and the public was not feasible for this secondary analysis, the original survey was conducted with rigorous ethical standards, and informed verbal consent was obtained from all participants.

### Data management

Data management for the current study is done using Stata version 15 and new variables are generated based on the research objectives. The dependent/outcome variables for this study are chosen from the urine and blood sample measurement section (Step 3) and the independent variables are selected from demographic and behavioral information (Step 1 and Step 2).

### Data access and anonymization

The original survey data, collected between January 2018 and Dec 2019 from the National Survey of Non-Communicable Disease STEPs (NCD STEPS) in Afghanistan, was accessed from July 7, 2021, to December 31, 2021, for the purpose of this study. The issue was coordinated formally and verbally with the relevant directorate within the Ministry of Public Health and the relevant authority within the WHO responsible for the survey. The dataset used in this study is not being updated.

Personal identifier information, including names, surnames, and phone numbers, was initially collected during the original survey to facilitate follow-up and ensure data accuracy. However, for the final dataset used in this secondary analysis, all personal identifiers were removed to de-identify the identity of the participants and ensure data privacy. The anonymization process followed standard data protection protocols, which included removing personal identifiers during the data cleaning and analysis phases. This ensures the confidentiality and privacy of the participants’ information.

### Ethical consideration

A formal approval from the Institutional Review Board (IRB) was obtained for the original survey (registration no. 43917/2017) and all ethical standards such as voluntary participation in the study were followed. Proper informed verbal consent was obtained from the study participants in the original survey and the response was documented in the questionnaires. Also, joint data quality monitoring visits were conducted with representatives from the IRB team. The current study maintained the confidentiality of the shared dataset and did not use it for any other purpose rather than this.

### Statistical methods

The Stata version 15 is used for the analysis. Survey setting is done using the PSU and step 3 weighting (wstep3) which was generated for the urine and blood sample testing section of the questionnaire (step3). Afterward, distributions and means of all the independent variables were calculated based on TC and BG levels to present the socio-demographic, behavioral characteristics, and physical measurement information of the participants. Moreover, in order to determine the association between dependent and independent variables, univariate linear regression was run for the general study population. Variables found to have a significant relation in the linear regression were further re-assessed by multivariable level using multiple linear regression to observe the linear relationship of these independent variables with the dependent (outcome) variable. The regression coefficients are presented with a 95% confidence level and the p-value<0.05 is considered statistically significant.

### Operational definition of variables

**Predictors/independent variables:**

- **Age:** The study includes participants aged 18 to 69 years old.
- **Age Category:** The age categories are taken from the original survey based on the Global Burden of Disease (GBD) [17]; there are three age categories for participants; 1) 18-29; 2) 30-44; and, 3) 45-69 considering categories for age in the original survey.
- **Gender:** Refers to respondents’ sex being 1) Male; or, 2) Female.
- **Education:** This variable was newly generated based on the International Standard Classification of Education (ISCED-2011). Four categories were considered for this variable; 1) No Education; 2) less than primary and primary education completed was combined in “Primary Education”; 3) secondary and high school completed was combined in “Secondary Education”; and 4) college/university and post graduate education was combined in “Tertiary Education”.
- **Marital Status:** In this study, marital status is classified into two categories; 0) Unmarried; and 1) Ever Married. Ever married includes all other categories including currently married, separated, widowed, and divorced.
- **Salt Intake:** The original question for this variable was “How often do you add **salt or a salty sauce such as soya sauce to your food** right before you eat it or as you are eating it” for which the response categories are re-defined from “always”, “often”, “sometimes”, “rarely” and “never” to 1) “Never”; 2) often, sometimes and rarely are combined into “Sometimes”; and 3) “Always”.
- **Any type of physical activities:** This variable in newly generated, it is the combination of 5 below variables:
  - “Whether participants’ work involve or they practically do vigorous-intensity activities or sports that cause a large increase in breathing or heart rate like [running or football] for at least 10 minutes continuously (2 variables included here); OR
  - Whether participants’ work involve or they practically do moderate-intensity activities or sports that cause a small increase in breathing or heart rate such as brisk walking, [cycling, swimming, volleyball] for at least 10 minutes continuously (2 variables included here); OR
  - Whether participant walks or uses a bicycle (pedal cycle) for at least 10 minutes continuously to get to and from places (1 variable included here)”

The response categories for this variable are 1) Yes; and 0) No.

- **Raised Blood Pressure (Hypertension):** This variable is newly generated from systolic and diastolic blood pressure measurement as well as from the question of “whether the respondent had been treated for raised blood pressure with drugs (medications) prescribed by a doctor or other health worker”. In order to ensure the accuracy of the data, 3 readings for systolic and diastolic blood pressure were recorded during the data collection process; however, in the analysis process for the original survey report, the average of the latest 2 readings was calculated. Following the same process; an average systolic blood pressure of =>140mm/hg and average diastolic blood pressure of =>90mm/hg was considered Raised Blood Pressure (Hypertension) and/or if the respondent was on treatment with hypertension drugs in the past two weeks; and the responses of 1) Yes; and 0) No were considered as its categories.
- **BMI (Body Mass Index):** This variable is also newly generated from height and weight measurement; it is calculated as weight (in kilogram) divided by height power to 2 (in meters).
- **BMI classification**
  - **Underweight:** This variable is based on the BMI classification of <18.5 kg/m^2^.
  - **Normal weight:** This variable is based on the BMI classification of between >=18.5 to <25 kg/m^2^.
  - **Overweight:** This variable is based on the BMI classification of between 25 to <30 kg/m^2^.
  - **Obesity:** This variable is based on the BMI classification of ≥30 kg/m^2.^

**Outcome/dependent variables:**

- Total Cholesterol Levels (participants’ fasting total cholesterol levels in mg/dl)
- Blood Glucose Levels (participants’ fasting blood glucose levels in mg/dl)

## Results

### Sample characteristics

**Fig 1:**
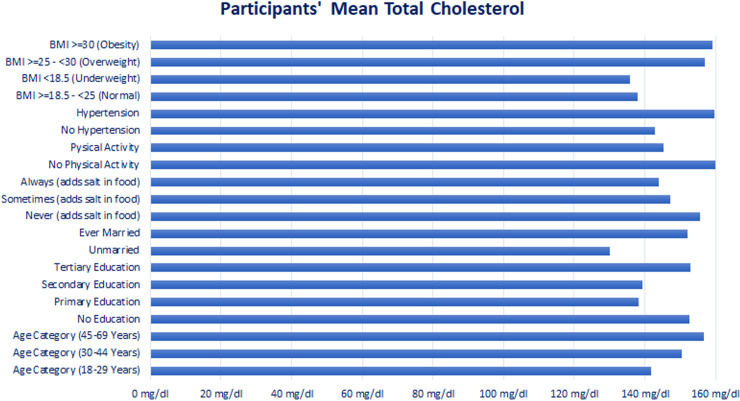
Participants’ Mean Total Cholesterol. Mean total cholesterol of the participants.

**Fig 2:**
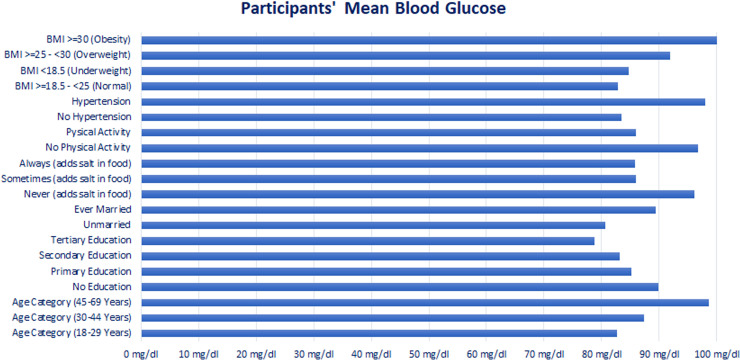
Participants’ Mean Blood Glucose. Mean blood glucose of the participants.

“Table 1” shows the mean level TC and BG, their distribution as well as univariate analysis using simple linear regression of total cholesterol level and blood glucose with sociodemographic, behavioral as well as BMI and High Blood pressure variables. Females, ever-married respondents, higher age categories, having hypertension, and higher BMI indicated increased mean levels of total cholesterol and blood glucose while the mean levels decreased with higher salt intake and physical activity “Figs 1–2”.

**Table 1:** Frequency & Mean Distribution of Total Cholesterol & Blood Glucose by different factors and univariate analysis using simple linear regression.

Categorical Independent Variables		Total Cholesterol (TC)							Blood Glucose (BG)						
		(n)	Mean TC (mg/dl)	Distribution %			Crude Regression		(n)	Mean BG (mg/dl)	Distribution %			Crude Regression	
				<200	200-239	>240	Coeff (95% CI)	p-value			<99	100-125	>126	Coeff (95% CI)	p-value
**Gender**	**Female**	1,754	156.7	81.2%	15.4%	3.4%	Rf		1,728	89.5	79.5%	10.8%	9.7%	Rf	
	**Male**	1,896	139.9	93.2%	4.9%	1.9%	-16.85 (-25.54 to -8.16)	<0.001	1,861	86.3	81.4%	12.1%	6.4%	-3.27 (-8.51 to 1.98)	0.221
**Age Categories**	**18-29**	1,358	141.7	89.7%	8.0%	2.4%	Rf		1,334	82.6	86.0%	10.1%	3.9%	Rf	
	**30-44**	1,055	150.6	87.2%	10.1%	2.7%	8.90 (3.64 to 14.15)	<0.001	1,043	87.4	82.8%	9.0%	8.2%	4.78 (0.38 to 9.17)	<0.05
	**45-69**	1,237	156.7	83.3%	13.6%	3.1%	14.99 (8.36 to 21.61)		1,212	98.6	66.7%	17.5%	15.9%	15.98 (12.30 to 19.67)	<0.001
**Education Categories**	**No Education**	2,030	152.5	84.8%	11.9%	3.3%	Rf		2,003	89.8	77.7%	12.9%	9.4%	Rf	
	**Primary Education**	626	138.2	91.3%	8.0%	0.8%	-14.30 (-21.45 to -7.14)	<0.001	613	85.2	83.6%	7.9%	8.5%	-4.64 (-10.68 to 1.41)	0.132
	**Secondary Education**	790	139.3	93.9%	5.3%	0.8%	-13.28 (-18.16 to -8.40)		771	83.2	85.8%	11.0%	3.2%	-6.61 (-10.64 to -2.59)	<0.01
	**Tertiary Education**	167	152.8	88.6%	4.0%	7.4%	0.27 (-9.77 to 10.30)	0.958	166	78.7	94.6%	2.3%	3.2%	-11.14 (-17.06 to -5.21)	<0.001
**Marital Status**	**Unmarried**	543	130.2	95.4%	3.8%	0.8%	Rf		532	80.7	92.3%	4.8%	3.0%	Rf	
	**Ever Married**	3,107	152.0	85.6%	11.4%	3.1%	21.76 (16.94 to 26.59)	<0.001	3,057	89.4	77.9%	12.9%	9.2%	8.79 (4.21 to 13.36)	<0.001
**Salt Intake**	**Never**	899	155.6	80.5%	16.0%	3.4%	Rf		890	96.2	71.8%	13.8%	14.4%	Rf	
	**Sometimes**	2,128	147.2	89.9%	8.2%	1.9%	-8.55 (-15.64 to -1.45)	<0.05	2,089	86.0	82.9%	11.1%	6.0%	-10.21 (-16.41 to -4.01)	<0.01
	**Always**	620	144.0	86.6%	9.5%	3.9%	-11.59 (-22.99 to -0.20)		607	85.8	81.5%	10.3%	8.2%	-10.41 (-17.16 to -3.67)	<0.05
**Any type of physical activity**	**No**	626	160.0	76.9%	18.6%	4.6%	Rf		615	96.7	72.3%	13.9%	13.8%	Rf	
	**Yes**	3,024	145.5	89.6%	8.2%	2.2%	-14.54 (-22.86 to -6.22)	<0.01	2,974	86.0	82.2%	11.0%	6.8%	-10.70 (-16.70 to -4.70)	<0.01
**Hypertension**	**No**	2,499	142.9	90.7%	7.2%	2.1%	Rf		2,464	83.5	86.3%	9.0%	4.7%	Rf	
	**Yes**	1,151	159.7	79.8%	16.3%	3.9%	16.80 (10.73 to 22.86)	<0.001	1,125	98.0	67.2%	17.1%	15.8%	14.51 (9.32 to 19.70)	<0.001
**BMI Classification**															
**≥18.5 to <25 (Normal)**		1,642	138.1	93.8%	5.1%	1.1%	Rf		1,616	82.8	86.1%	9.5%	4.4%	Rf	
**<18.5 (Underweight)**		243	135.8	90.7%	8.9%	0.4%	-2.28 (-12.35 to 7.78)	0.656	235	84.7	85.5%	8.8%	5.7%	1.87 (-4.20 to 7.94)	0.544
****≥**25 to <30 (Overweight)**		1,003	157.0	83.3%	13.0%	3.7%	18.95 (14.35 to 23.56)	<0.001	987	91.9	77.3%	12.6%	10.1%	9.11 (5.35 to 12.87)	<0.001
****≥**30 (Obesity)**		586	159.2	80.2%	15.8%	4.1%	21.11 (14.95 to 27.27)		575	101.8	62.1%	18.1%	19.8%	18.99 (10.77 to 27.21)	
**Numerical Dependent Variables**															
**Age**							0.42 (0.21 to 0.63)	<0.001						0.48 (0.36 to 0.59)	<0.001
**BMI (Body Mass Indiex)**							1.25 (0.73 to 1.77)	<0.001						0.89 (0.39 to 1.38)	<0.01

### Factors associated with total cholesterol and blood glucose:

#### Univariate analysis (simple linear regression).

**Fig 3:**
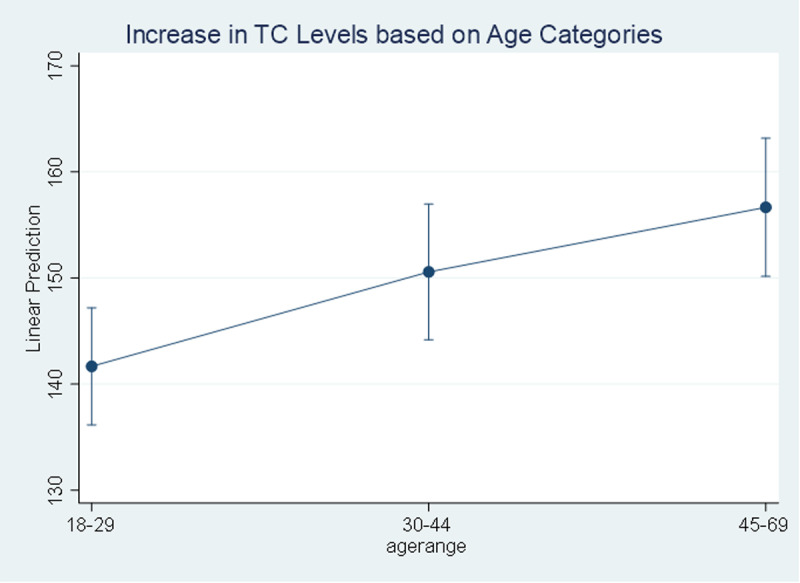
Increase in Total Cholesterol level based on Age Categories. Level of total cholesterol increasing based on age.

**Fig 4:**
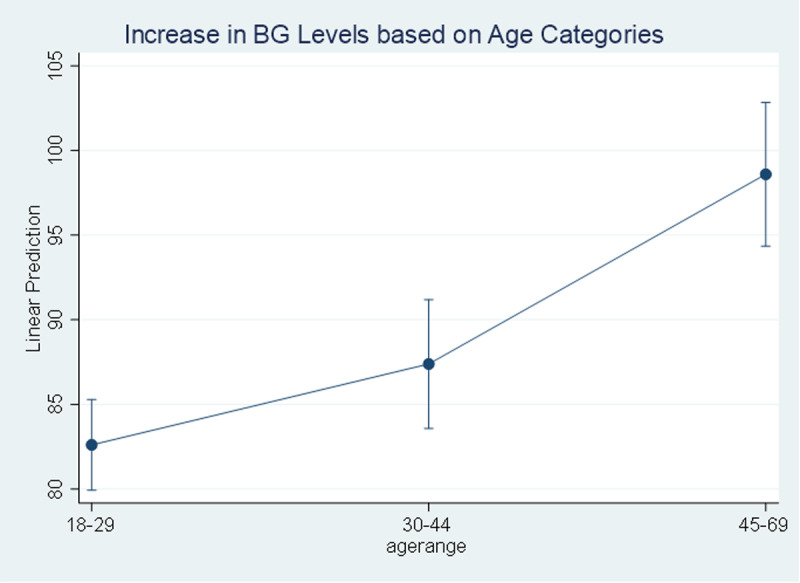
Increase in Blood Glucose level based on Age Categories. Level of blood glucose increasing based on age.

The result of univariate analysis using simple linear regression indicates that there are significant linear relationship between all the listed independent variables with the TC and BG levels (p-values<0.05). Age, marital status, high blood pressure, and BMI are positively associated with TC and BG levels while education, salt intake, and any type of physical activity are negatively associated with the TC and BG levels. The study found that with an increase of one year in age, the total cholesterol levels increase by 0.42 mg/dl [95% CI (0.21 to 0.63)] while blood glucose levels increase by 0.48 mg/dl [95% CI (0.36 to 0.59)], a 0.42 mg/dl increase in total cholesterol per year can contribute to a higher risk of cardiovascular diseases over time, while a 0.48 mg/dl increase in blood glucose per year may indicate a growing risk of developing diabetes. Also, the total cholesterol levels increase by 15.0 mg/dl [95% CI (8.36 to 21.61)] for people aged between 45-69 years compared to younger people aged between 18 to 29 years old while this figure is 16.0 mg/dl [95% CI (12.30 to 19.67)] for blood glucose levels “Figs 3–4”.

**Fig 5:**
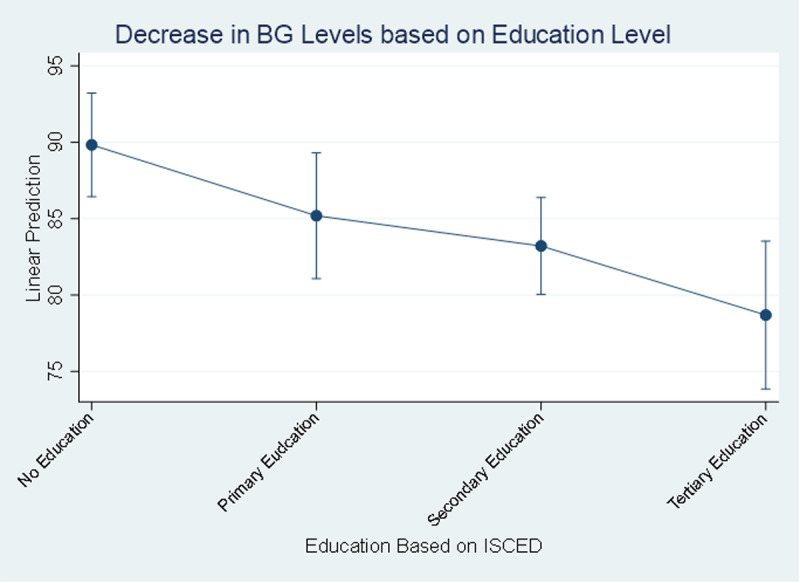
Decrease in Blood Glucose level based on Education Level. Level of total cholesterol decreasing based on salt intake.

Moreover, the TC level decreases by 14.3 mg/dl [95% CI (-21.45 to -7.14)] in people having primary education and by 13.3 mg/dl [95% CI (-18.16 to -8.40)] in people having secondary education compared to no education category, while the tertiary education category was not significantly associated with TC levels. The BG level decreases by 6.6 mg/dl [95% CI (-10.64 to -2.59)] in people having secondary education and by 11.1 mg/dl [95% CI (-17.06 to -5.21)] in people having tertiary education compared to the no education category, while the primary education category was not significantly associated with BG levels “Fig 5”. All total cholesterol and blood glucose measurements are reported in mg/dl for consistency.

As for marital status, there is an increase of 21.8 mg/dl [95% CI (16.94 to 26.59)] and 8.8 mg/dl [95% CI (4.21 to 13.36)] in TC and BG levels respectively in ever-married people compared to unmarried ones. This means that ever-married individuals have higher TC and BG levels compared to unmarried individuals.

**Fig 6:**
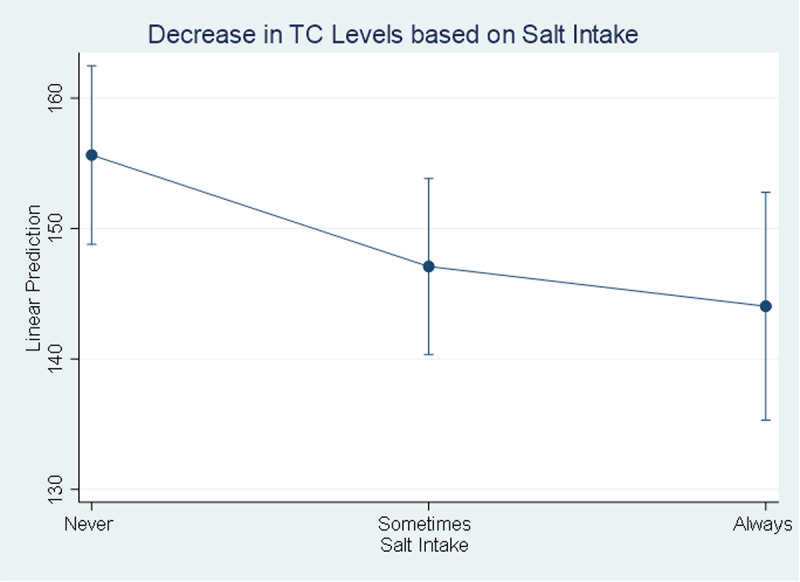
Decrease in Total Cholesterol level based on Salt Intake. Level of total cholesterol decreasing based on salt intake.

Considering the behavioral factors; the level of TC and BG decreases by 11.6 mg/dl [95% CI (-22.99 to -0.20)] and 10.4 mg/dl [95% CI (-17.16 to -3.67)] when people always take salt “Fig 6”; also, participating in any type of physical activity in a week decreases the TC and BG levels by 14.5 mg/dl [95% CI (-22.86 to -6.22)] and 10.7 mg/dl [95% CI (-16.70 to -4.70)] respectively.

Hypertension increases the levels of TC and BG by 16.8 mg/dl [95% CI (10.73 to 22.86)] and 14.5 mg/dl [95% CI (9.32 to 19.70)]; also, one unit increase in Quetelet Index (BMI) increases the TC and BG levels by 1.3 mg/dl [95% CI (0.73 to 1.77)] and 0.9 mg/dl [95% CI (0.39 to 1.38)] respectively. Moreover, considering BMI classification; being overweight and having obesity increases the levels of TC by 19.0 mg/dl [95% CI (14.35 to 23.56)] and 21.1 mg/dl [95% CI (14.95 to 27.27)] and increases the level of BG by 9.1 mg/dl [95% CI (5.35 to 12.87)] and 19.0 mg/dl [95% CI (10.77 to 27.21)] respectively.

#### Multivariable analysis using multiple linear regression.

The result showed that when controlling for all independent variables included in this analysis; it seemed that the association between total cholesterol was significant with the male category of gender (Coeff -8.42, 95% CI: -16.49 to -0.34), the age category of 45-69 years (Coeff 6.07, 95% CI: 0.09 to 12.04), ever married category of marital status (Coeff 9.23, 95% CI: 4.32 to 14.14), having hypertension (Coeff 11.59, 95% CI: 6.49 to 16.68), overweight or BMI of ≥25 to <30 (Coeff 11.63, 95% CI: 6.09 to 17.18), and Obesity or BMI of ≥30 (Coeff 12.34, 95% CI: 6.59 to 18.10) and apart from gender category all other variables had positive associations with total cholesterol; while the association education, salt intake, physical activity, and underweight category of BMI with total cholesterol were no longer significant in our model.

As for blood glucose, the association was significant with age category of 45-69 years of age (Coeff 8.23, 95% CI: 3.92 to 12.53), having tertiary education level (Coeff -7.49, 95% CI: -12.96 to -2.03), taking salt always (Coeff -8.63, 95% CI: -14.99 to -2.27), participating in any type of physical activity (Coeff -8.12, 95% CI: -14.01 to -2.23), having hypertension (Coeff 8.40, 95% CI: 3.51 to 13.30), overweight or BMI of ≥25 to <30 (Coeff 4.80, 95% CI: 1.14 to 8.46), and Obesity or BMI of ≥30 (Coeff 13.44, 95% CI: 6.99 to 19.88); while the association with gender, other categories of age and education, other categories of salt intake and BMI, and marital status were no longer significant in our model.

Worth mentioning that there was no multicollinearity among the independent variables as the Variance Inflation Factor (VIF) was less than 4 (1.1) “Table 2”.

**Table 2:** Adjusted Regression Coefficients (95%CI) by different independent variables.

Categorical Independent Variables		Adjusted Regression TC		Adjusted Regression BG	
		Coeff (95% CI)	p-value	Coeff (95% CI)	p-value
**Gender**	Female	Rf		Rf	
	Male	-8.42 (-16.49 to -0.34)	<0.05	2.93 (-2.52 to 8.37)	0.291
**Age Categories**	18-29	Rf		Rf	
	30-44	2.57 (-2.44 to 7.59)	0.313	0.76 (-3.09 to 4.61)	0.697
	45-69	6.07 (0.09 to 12.04)	<0.05	8.23 (3.92 to 12.53)	<0.001
**Education Categories**	No Education	Rf		Rf	
	Primary Education	-6.31 (-12.90 to 0.29)	0.061	-3.65 (-10.47 to 3.17)	0.293
	Secondary Education	0.05 (-4.83 to 4.93)	0.983	-3.10 (-8.11 to 1.90)	0.223
	Tertiary Education	10.02 (-0.39 to 20.43)	0.059	-7.49 (-12.96 to -2.03)	<0.01
**Marital Status**	Unmarried	Rf		Rf	
	Ever Married	9.23 (4.32 to 14.14)	<0.001	1.28 (-4.07 to 6.64)	0.637
**Salt Intake**	Never	Rf		Rf	
	Sometimes	-1.44 (-8.32 to 5.44)	0.681	-6.86 (-13.83 to 0.11)	0.054
	Always	-8.57 (-17.49 to 0.35)	0.060	-8.63 (-14.99 to -2.27)	<0.01
**Any type of physical activity**	No	Rf		Rf	
	Yes	-4.60 (-11.28 to 2.09)	0.177	-8.12 (-14.01 to -2.23)	<0.01
**Hypertension**	No	Rf		Rf	
	Yes	11.59 (6.49 to 16.68)	<0.001	8.40 (3.51 to 13.30)	<0.01
**BMI Classes**	≥18.5 to <25 (Normal)	Rf		Rf	
	<18.5 (Underweight)	-1.84 (-11.34 to 7.67)	0.704	2.81 (-3.28 to 8.88)	0.365
	≥25 to <30 (Overweight)	11.63 (6.09 to 17.18)	<0.001	4.80 (1.14 to 8.46)	<0.05
	≥30 (Obesity)	12.34 (6.59 to 18.10)	<0.001	13.44 (6.99 to 19.88)	<0.001

## Discussion

The purpose of this study was to determine the associated factors of total cholesterol and blood glucose for people aged between 18-69 years old for which the results of bi-variate linear regression showed significant association (p-value <0.05) between age, marital status, high blood pressure, BMI, education, salt intake, any type of physical activity and total cholesterol as well as with blood glucose.

The study results concerning associations of factors such as education, socio-economic situations, hypertension, physical activity, and BMI are almost consistent with most of similar studies conducted in other countries; however, there were also differences in some factors, i.e., age. In our study higher age groups had a positive association with total cholesterol while most of studies concluded that total cholesterol decreases by each year of age in older people compared to younger people while the level of decrease differs among men and women [18–22] as in one of the study published in PubMed journal; the linear regression analysis result on men born between 1900 to 1920 from Dutch town in the years of 1977, 1978, 1985, 1990, and 1993 determines that total cholesterol decreased by 0.04 mmol/l (1.55 mg/dl) by each year of age [23]. Also, total cholesterol increases by age in women more than men due to a decline in estrogen hormone by the start of menopause [24,25]. On the other hand; the association between age and fasting blood glucose is complex and still under debate since many other factors can also affect the increase in blood glucose apart from age [26]

Considering the association between total cholesterol and socio-economic factors; our study result found that education is negatively associated with total cholesterol which is consistent with a cohort study on total cholesterol and HDL-cholesterol in relation to education status in Greek adults between the time frame of 1994 – 98; the study indicates that total cholesterol is inversely associated with the medium and high level of education in both women and men using multiple linear regression between total cholesterol and education level controlling for age. Worth mentioning that the education category is classified almost the same as the classification considered in our study. Moreover, the decrease in the coefficient ratio for the said association was more evident in women than in men [27]. Also, in another study on the impact of traditional and novel risk factors on the relationship between socioeconomic status and incidents of cardiovascular events; increased levels of education and income decreased the median level of total cholesterol as well as other lipids excluding high-density lipoprotein (HDL) on which the opposite occurred (P-value<0.001) [28].

The result of our study on the positive association of hypertension with total cholesterol is also consistent with studies in other countries. According to a Framingham study on the prevalence of high cholesterol levels in hypertensive patients; although both elevated levels of cholesterol and high blood pressure were risk factors for coronary heart disease, there was not a strong correlation between blood pressure and serum cholesterol. As well as more than half of patients with hypertension or requiring treatment for hypertension, also had lipid abnormality [29].

Moreover, High blood pressure and diabetes are comorbidities and combined risk factors for life-threatening cardiovascular disease (CVD) based on the review article on diabetes, hypertension, and cardiovascular disease: clinical insights and vascular mechanisms. The review also states that people with high blood pressure have an increased risk of developing diabetes compared to those with typical blood pressure [30]. Also, another study on hypertension and diabetes millitus found that hypertension and diabetes millitus predict each other overtime [31]. Our study also found that hypertension is positively associated with blood glucose while we considered high blood pressure as well as 2 weeks on treatment for hypertension as hypertension in our study.

Our study found that there is a negative association between salt intake by people who always add salt to their food and total cholesterol as well as with blood glucose and salt intake; however, it is worth mentioning that no measurement had been applied to how much salt was being taken by participants in the original survey. And looking into other studies conducted on this matter; there are many articles indicating the side effects of salt intake on hypertension in general and hypertension in type II of diabetes [32–36]; however, functional salt intake has been found effective in decreasing total cholesterol in rats [37,38]. Studies also suggest that a large reduction in sodium or salt intake increases total cholesterol [39–41]; while moderate reduction tends to no significant increase in total cholesterol [40,42]. Another study on the effect of dietary sodium intake on blood lipids concluded that changes in dietary sodium intake over the range of 50 to 150 mmol/d did not affect blood lipid concentrations [43].

On the other hand, the result of a study on the association between dietary salt and plasma glucose, insulin, and hemoglobin A_1c_ levels among type II diabetes patients in eastern China, indicates that higher dietary salt (>8 g/day) increased Fasting Plasma Glucose (FPG) by 2.3 mmol/l (41.44 mg/dl) compared to lower dietary salt. In addition, the study result also showed that high blood lipids were also significantly related to increased dietary salt intake [44]. They hypothesize that dietary salt is a key factor in increasing the feeling of thirst, resulting in a greater intake of fluid drinks [45]. A difference of 1 g/day salt intake was positively associated with a difference of 100 g/day total fluid and 27 g/day sugar-sweetened soft drink consumption [46]. Moreover, some studies found that there is no significant association between salt intake and blood glucose while the level of increase or decrease in blood glucose in relation to lower or higher salt intake differed [47,48].

Physical activity has been found negatively associated with total cholesterol and blood glucose in our study which is consistent with most studies that suggest that an increase in physical activity is negatively associated with blood glucose as well as serum total cholesterol especially triglycerides [49–51]. While there are some studies that show no significant relationship between the substitution of sedentary activities with moderate-intensity physical activities and cardio-metabolic risk biomarkers; some others show a decrease in fasting blood glucose and total cholesterol levels with the above-mentioned substitution [52–54].

BMI and obesity were found to be positively associated with total cholesterol and blood glucose in our study which again shows consistency with other studies. Based on study on “correlation between BMI and fasting total blood cholesterol level among undergraduate students in Malaysia”, there was a significant correlation between BMI and fasting total cholesterol in overall participants while the correlation was not significant for females [55]. Also, in other studies on correlations between BMI and fasting blood lipids on health obese [56] and morbidly obese participants [57]; triglycerides are found to have a positive association with BMI while the associations are different for other types of lipids. Moreover, according to a cohort study “Hypertension and Diabetes Mellitus”, BMI and weight gain were significant predictors of both incident hypertension and diabetes millitus [29]. Also, results from some studies show that BMI has positive correlations with blood glucose [58,59] while the correlation was weak for males and strong for females [60].

To build upon the findings of this study, future research studies could explore the longitudinal effects of lifestyle interventions on total cholesterol and blood glucose levels, investigate the genetic predispositions influencing these biomarkers, and examine the impact of socio-economic factors on the management and prevention of hypercholesterolemia and hyperglycemia in diverse populations. Also, future analytical studies are recommended to find the causal relationship between dependent and independent variables.

## Limitations

The findings of this study should be interpreted considering some of the limitations of the study as well. First, the original study (NCD STEPs survey) had cross-sectional survey settings; thus, causal inferences should not be made based on these findings. Secondly, we faced major challenges during the analysis of data due to data quality in some variables and even had to drop some of the variables such as smoking, occupation, and years of education. There were more than 3,950 participants among which only 350 were current smokers, the variable was not significant with most of the other variables including total cholesterol and blood glucose with a wider confidence interval while there were overlaps in the categories of occupation variable.

As for Education variables, there were two variables within the dataset; 1) Years of Education; and 2) Education Level. There was no consistency between these two variables in some of the observations, e.g., in the “Years of Education” variable, a person had 12 years of education while in the “Education Level” variable “no formal schooling” category was selected; thus, we had to select only one of these variables and we moved forward with our analysis considering only the Education Level of participants.

Also, worth mentioning that no measurement was performed on the quantity of salt intake in the original survey and only participants’ responses on how often they add salt or salty sauces to their food, are considered as a variable for salt intake in the current study. This limitation should also be taken into account when interpreting the results, as the actual salt consumption may vary significantly among participants.

## Conclusion

The findings of this study show that total cholesterol and blood glucose levels increase in people of older age categories, ever married people, people with hypertension, and those who are overweight and obese; while they decrease in people with higher education, those who consistently add salt or salty sauces to their food, and those who engage in physical exercise. These results suggest that older adults, ever married individuals, and those with hypertension or higher BMI might be at greater risk for cardiovascular diseases and diabetes. Conversely, higher education, regular physical activity, and consistent salt intake may offer protective benefits against these conditions.

In order to address these challenges, practical solutions include increasing public awareness about regular health check-ups, promoting healthier lifestyles through education, improving access to healthcare services for managing hypertension and diabetes, and developing policies for healthier food options and more public spaces for exercise. These measures can mitigate the challenges identified in this study and improve public health outcomes.

## References

[1] Lee Y, Siddiqui W. Cholesterol Levels. In: StatPearls [Internet]. Treasure Island (FL): StatPearls Publishing; 2022 [cited 2022 Nov 10]. Available from: http://www.ncbi.nlm.nih.gov/books/NBK542294/

[2] Blood Sugar [Internet]. National Library of Medicine; [cited 2022 Dec 5]. Available from: https://medlineplus.gov/bloodsugar.html

[3] Indicator Metadata Registry Details [Internet]. 2022 [cited 2022 Dec 5]. Available from: https://www.who.int/data/gho/indicator-metadata-registry/imr-details/2380

[4] Cleveland Clinic [Internet]. [cited 2022 Dec 5]. Understanding Cholesterol Levels and Numbers. Available from: https://my.clevelandclinic.org/health/articles/11920-cholesterol-numbers-what-do-they-mean

[5] CDC. Centers for Disease Control and Prevention. 2022 [cited 2022 Nov 16]. Diabetes Testing. Available from: https://www.cdc.gov/diabetes/basics/getting-tested.html

[6] Mean Total Cholesterol (crude estimate) [Internet]. [cited 2022 Nov 16]. Available from: https://www.who.int/data/gho/data/indicators/indicator-details/GHO/mean-total-cholesterol-(crude-estimate)

[7] Danaei G, Finucane M, Lu Y, Singh G, Cowan M, Paciorek C, et al. National, regional, and global trends in fasting plasma glucose and diabetes prevalence since 1980: systematic analysis of health examination surveys and epidemiological studies with 370 country-years and 2.7 million participants. Lancet. 2011;378(9785):31–40. doi: 10.1016/S0140-6736(11)60679-0 21705069

[8] Afghanistan - STEPS 2018 [Internet]. [cited 2022 Dec 4]. Available from: https://extranet.who.int/ncdsmicrodata/index.php/catalog/782

[9] Martin MJ, Hulley SB, Browner WS, Kuller LH, Wentworth D. Serum cholesterol, blood pressure, and mortality: implications from a cohort of 361,662 men. Lancet. 1986;2(8513):933–6. doi: 10.1016/s0140-6736(86)90597-0 2877128

[10] Ezzati M. Comparative quantification of health risks: Sexual and reproductive health. 2004.

[11] Tisseverasinghe A, Lim S, Greenwood C, Urowitz M, Gladman D, Fortin PR. Association between serum total cholesterol level and renal outcome in systemic lupus erythematosus. Arthritis Rheum. 2006;54(7):2211–9. doi: 10.1002/art.21929 16802357

[12] World Health Organization. Global status report on noncommunicable diseases 2010 [Internet]. World Health Organization; 2011 [cited 2023 Jun 18]. Available from: https://apps.who.int/iris/handle/10665/44579

[13] GBD 2015 Risk Factors Collaborators. Global, regional, and national comparative risk assessment of 79 behavioural, environmental and occupational, and metabolic risks or clusters of risks, 1990-2015: a systematic analysis for the Global Burden of Disease Study 2015. Lancet. 2016;388(10053):1659–724. doi: 10.1016/S0140-6736(16)31679-8 27733284 PMC5388856

[14] Harlan M, Krumholz MD. Worldwide Trends in Cholesterol Levels. NEJM J Watch [Internet]. 2020 Jun 17 [cited 2023 Jun 18];2020. Available from: https://www.jwatch.org/NA51768/2020/06/17/worldwide-trends-cholesterol-levels

[15] Saeedi P, Petersohn I, Salpea P, Malanda B, Karuranga S, Unwin N, et al. Global and regional diabetes prevalence estimates for 2019 and projections for 2030 and 2045: Results from the International Diabetes Federation Diabetes Atlas, 9th edition. Diabetes Research and Clinical Practice. 2019;157:107843. doi: 10.1016/j.diabres.2019.107843 31518657

[16] Liu J, Bai R, Chai Z, Cooper M, Zimmet P, Zhang L. Low- and middle-income countries demonstrate rapid growth of type 2 diabetes: an analysis based on Global Burden of Disease 1990–2019 data. Diabetologia. 2022;65(8):1339–52. doi: 10.1007/s00125-022-05756-5 35587275 PMC9118183

[17] Manual [Internet]. [cited 2023 Mar 4]. Available from: https://www.who.int/teams/noncommunicable-diseases/surveillance/systems-tools/steps/manuals

[18] Carroll MD, Lacher DA, Sorlie PD, Cleeman JI, Gordon DJ, Wolz M, et al. Trends in serum lipids and lipoproteins of adults, 1960-2002. JAMA. 2005;294(14):1773–81. doi: 10.1001/jama.294.14.1773 16219880

[19] Ferrara A, Barrett-Connor E, Shan J. Total, LDL, and HDL cholesterol decrease with age in older men and women. The Rancho Bernardo Study 1984-1994. Circulation. 1997;96(1):37–43. 9236414 10.1161/01.cir.96.1.37

[20] Wilson PW, Anderson KM, Harris T, Kannel WB, Castelli WP. Determinants of change in total cholesterol and HDL-C with age: the Framingham Study. J Gerontol. 1994;49(6):M252–7. doi: 10.1093/geronj/49.6.m252 7963277

[21] Ettinger WH, Wahl PW, Kuller LH, Bush TL, Tracy RP, Manolio TA, et al. Lipoprotein lipids in older people. Results from the Cardiovascular Health Study. The CHS Collaborative Research Group. Circulation. 1992;86(3):858–69. doi: 10.1161/01.cir.86.3.858 1516198

[22] Garry PJ, Hunt WC, Koehler KM, VanderJagt DJ, Vellas BJ. Longitudinal study of dietary intakes and plasma lipids in healthy elderly men and women. Am J Clin Nutr. 1992;55(3):682–8. doi: 10.1093/ajcn/55.3.682 1550044

[23] Mp W, Ej F, D K. Age-related changes in total and high-density-lipoprotein cholesterol in elderly Dutch men. Am J Public Health [Internet]. 1996 Jun [cited 2022 Dec 4];86(6). Available from: https://pubmed.ncbi.nlm.nih.gov/8659652/. 10.2105/ajph.86.6.798 PMC1380397 8659652

[24] Stevenson JC, Crook D, Godsland IF. Influence of age and menopause on serum lipids and lipoproteins in healthy women. Atherosclerosis. 1993;98(1):83–90. 8457253 10.1016/0021-9150(93)90225-j

[25] Matthews KA, Crawford SL, Chae CU, Everson-Rose SA, Sowers MF, Sternfeld B, et al. Are changes in cardiovascular disease risk factors in midlife women due to chronological aging or to the menopausal transition?. J Am Coll Cardiol. 2009;54(25):2366–73. doi: 10.1016/j.jacc.2009.10.009 20082925 PMC2856606

[26] Meigs JB, Muller DC, Nathan DM, Blake DR, Andres R. The natural history of progression from normal glucose tolerance to type 2 diabetes in the Baltimore Longitudinal Study of Aging. Diabetes. 2003;52(6):1475–84. 12765960 10.2337/diabetes.52.6.1475

[27] Benetou V, Chloptsios Y, Zavitsanos X, Karalis D, Naska A, Trichopoulou A. Total cholesterol and HDL-cholesterol in relation to socioeconomic status in a sample of 11,645 Greek adults: the EPIC study in Greece. Scand J Public Health. 2000;28(4):260–5. 11228112

[28] Albert MA, Glynn RJ, Buring J, Ridker PM. Impact of traditional and novel risk factors on the relationship between socioeconomic status and incident cardiovascular events. Circulation. 2006;114(24):2619–26. doi: 10.1161/CIRCULATIONAHA.106.660043 17116764

[29] Castelli W, Anderson K. A population at risk. Prevalence of high cholesterol levels in hypertensive patients in the Framingham Study. American Journal of Medicine. 1986;80(2A):23–32. 10.1016/0002-9343(86)90157-9 3946458

[30] Petrie JR, Guzik TJ, Touyz RM. Diabetes, Hypertension, and Cardiovascular Disease: Clinical Insights and Vascular Mechanisms. Can J Cardiol. 2018;34(5):575–84. doi: 10.1016/j.cjca.2017.12.005 29459239 PMC5953551

[31] Tsimihodimos V, Gonzalez-Villalpando C, Meigs J, Ferrannini E. Hypertension and diabetes mellitus. Hypertension. 2018;71(3):422–8. 29335249 10.1161/HYPERTENSIONAHA.117.10546 PMC5877818

[32] Grillo A, Salvi L, Coruzzi P, Salvi P, Parati G. Sodium intake and hypertension. Nutrients. 2019;11(9):1970. doi: 10.3390/nu11091970 31438636 PMC6770596

[33] Intersalt Cooperative Research Group. Intersalt: an international study of electrolyte excretion and blood pressure. Results for 24 hour urinary sodium and potassium excretion. BMJ. 1988;297(6644):319–28. 3416162 10.1136/bmj.297.6644.319 PMC1834069

[34] He FJ, MacGregor GA. Effect of modest salt reduction on blood pressure: a meta-analysis of randomized trials. Implications for public health. J Hum Hypertens. 2002;16(11):761–70. doi: 10.1038/sj.jhh.1001459 12444537

[35] Denton D, Weisinger R, Mundy NI, Wickings EJ, Dixson A, Moisson P, et al. The effect of increased salt intake on blood pressure of chimpanzees. Nat Med. 1995;1(10):1009–16. doi: 10.1038/nm1095-1009 7489355

[36] Mente A, O’Donnell MJ, Rangarajan S, McQueen MJ, Poirier P, Wielgosz A, et al. Association of urinary sodium and potassium excretion with blood pressure. N Engl J Med. 2014;371(7):601–11. doi: 10.1056/NEJMoa1311989 25119606

[37] Takagi Y, Sugimoto T, Kobayashi M, Shirai M, Asai F. High-Salt Intake Ameliorates Hyperglycemia and Insulin Resistance in WBN/Kob-Leprfa/fa Rats: A New Model of Type 2 Diabetes Mellitus. J Diabetes Res. 2018;2018:3671892. doi: 10.1155/2018/3671892 29744365 PMC5884204

[38] Journal of the Korean Society of Food Science and Nutrition [Internet]. [cited 2022 Dec 5]. Available from: https://www.e-jkfn.org/journal/view.html?uid=6368&vmd=Full

[39] Egan BM, Lackland DT. Biochemical and metabolic effects of very-low-salt diets. Am J Med Sci. 2000;320(4):233–9. doi: 10.1097/00000441-200010000-00002 11061347

[40] Ruppert M, Overlack A, Kolloch R, Kraft K, Göbel B, Stumpe KO. Neurohormonal and metabolic effects of severe and moderate salt restriction in non-obese normotensive adults. J Hypertens. 1993;11(7):743–9. doi: 10.1097/00004872-199307000-00010 8228194

[41] Del Río A, Rodríguez-Villamil JL. Metabolic effects of strict salt restriction in essential hypertensive patients. J Intern Med. 1993;233(5):409–14. doi: 10.1111/j.1365-2796.1993.tb00692.x 8487006

[42] Grey A, Braatvedt G, Holdaway I. Moderate dietary salt restriction does not alter insulin resistance or serum lipids in normal men. Am J Hypertens. 1996;9(4 Pt 1):317–22. doi: 10.1016/0895-7061(95)00390-8 8722434

[43] Harsha DW, Sacks FM, Obarzanek E, Svetkey LP, Lin PH, Bray GA, et al. Effect of dietary sodium intake on blood lipids. Hypertension. 2004;43(2):393–8. 14707154 10.1161/01.HYP.0000113046.83819.a2

[44] Lin Y, Chattopadhyay K, Yang X, Li J, Chen Y, Zhou Y. Association between dietary salt and plasma glucose, insulin and hemoglobin A1c levels among type 2 diabetes patients in Eastern China. Diabetes Metab Syndr Obes. 2021;14:4811–8. 34984013 10.2147/DMSO.S338915 PMC8699762

[45] He FJ, Markandu ND, Sagnella GA, MacGregor GA. Effect of salt intake on renal excretion of water in humans. Hypertension. 2001;38(3):317–20. doi: 10.1161/01.hyp.38.3.317 11566897

[46] He F, Marrero N, MacGregor G. Salt intake is related to soft drink consumption in children and adolescents: a link to obesity? Hypertension. 2008;51(3):629–34. 18287345 10.1161/HYPERTENSIONAHA.107.100990

[47] Shen Y, Shi Y, Cui J, He H, Ren S. Effects of dietary salt intake restriction on blood glucose levels: a meta-analysis of crossover study. Nutr Res Pract [Internet]. 2022 Nov 10 [cited 2023 Mar 11];17. Available from: https://www.e-nrp.org/Synapse/10.4162/nrp.2023.17.e9 10.4162/nrp.2023.17.3.387 PMC10232203 37266115

[48] Lin Y, Mei Q, Qian X, He T. Salt consumption and the risk of chronic diseases among Chinese adults in Ningbo city. Nutr J. 2020;19(1):9. doi: 10.1186/s12937-020-0521-8 31996216 PMC6990556

[49] Sabaka P, Kruzliak P, Balaz D, Komornikova A, Celovska D, Cammarota G. Effect of short term aerobic exercise on fasting and postprandial lipoprotein subfractions in healthy sedentary men. Lipids in Health and Disease. 2015;14:151. 26607422 10.1186/s12944-015-0148-5 PMC4658794

[50] Omar A, Husain MN, Jamil AT, Nor NSM, Ambak R, Fazliana M, et al. Effect of physical activity on fasting blood glucose and lipid profile among low income housewives in the MyBFF@home study. BMC Womens Health. 2018;18(Suppl 1):103. doi: 10.1186/s12905-018-0598-9 30066645 PMC6069292

[51] Salse-Batán J, Pérez BV, Sánchez-Lastra MA, Pérez CA. The effects of exercise and intermittent fasting on health: a systematic review.

[52] Ekblom-Bak E, Ekblom Ö, Bolam KA, Ekblom B, Bergström G, Börjesson M. SCAPIS Pilot Study: Sitness, Fitness and Fatness - Is Sedentary Time Substitution by Physical Activity Equally Important for Everyone’s Markers of Glucose Regulation?. J Phys Act Health. 2016;13(7):697–703. doi: 10.1123/jpah.2015-0611 26900674

[53] Buman MP, Winkler EAH, Kurka JM, Hekler EB, Baldwin CM, Owen N, et al. Reallocating time to sleep, sedentary behaviors, or active behaviors: associations with cardiovascular disease risk biomarkers, NHANES 2005-2006. Am J Epidemiol. 2014;179(3):323–34. doi: 10.1093/aje/kwt292 24318278

[54] Healy G, Winkler E, Owen N, Anuradha S, Dunstan D. Replacing sitting time with standing or stepping: associations with cardio-metabolic risk biomarkers. Eur Heart J. 2015;36(39):2643–9. 26228867 10.1093/eurheartj/ehv308

[55] Correlation Between Body Mass Index (BMI) and Fasting Total Blood Cholesterol Level among Undergraduate Students [Internet]. [cited 2023 Jan 6]. Available from: https://scialert.net/abstract/?doi=pjn.2016.873.877

[56] Telles S, Pal S, Sharma S, Singh A, Kala N, Balkrishna A. The association between the lipid profile and fasting blood glucose with weight related outcomes in healthy obese adults. BMC Res Notes [Internet]. 2018 [cited 2023 Jan 6];11. Available from: https://www.ncbi.nlm.nih.gov/pmc/articles/PMC6000929/. 10.1186/s13104-018-3485-4 PMC6000929 29898771

[57] Shamai L, Lurix E, Shen M, Novaro GM, Szomstein S, Rosenthal R, et al. Association of body mass index and lipid profiles: evaluation of a broad spectrum of body mass index patients including the morbidly obese. Obes Surg. 2011;21(1):42–7. doi: 10.1007/s11695-010-0170-7 20563664

[58] Agrawal N, Agrawal M, Kumari T, Kumar S. Correlation between body mass index and blood glucose levels in Jharkhand population. 2017;4(8).

[59] Doustjalali SR, Sabet NS, Amm AA. Correlation between Body Mass Index (BMI) and Fasting Blood Glucose (FBG) Level among Malaysian Adults Age 40-60.

[60] Innocent O, ThankGod O, Sandra E, Josiah I. Correlation between body mass index and blood glucose levels among some Nigerian undergraduates. HOAJ Biology. 2013;2(1):4.

